# Scaling the extinction vortex: Body size as a predictor of population dynamics close to extinction events

**DOI:** 10.1002/ece3.7555

**Published:** 2021-05-02

**Authors:** Nathan F. Williams, Louise McRae, Robin Freeman, Pol Capdevila, Christopher F. Clements

**Affiliations:** ^1^ School of Biological Sciences University of Bristol Bristol UK; ^2^ Institute of Zoology Zoological Society of London London UK

**Keywords:** body size, extinction vortex, pace of life, population dynamics, population extinction

## Abstract

1. Mutual reinforcement between abiotic and biotic factors can drive small populations into a catastrophic downward spiral to extinction—a process known as the “extinction vortex.” However, empirical studies investigating extinction dynamics in relation to species' traits have been lacking.
2. We assembled a database of 35 vertebrate populations monitored to extirpation over a period of at least ten years, represented by 32 different species, including 25 birds, five mammals, and two reptiles. We supplemented these population time series with species‐specific mean adult body size to investigate whether this key intrinsic trait affects the dynamics of populations declining toward extinction.
3. We performed three analyses to quantify the effects of adult body size on three characteristics of population dynamics: time to extinction, population growth rate, and residual variability in population growth rate.
4. Our results provide support for the existence of extinction vortex dynamics in extirpated populations. We show that populations typically decline nonlinearly to extinction, while both the rate of population decline and variability in population growth rate increase as extinction is approached. Our results also suggest that smaller‐bodied species are particularly prone to the extinction vortex, with larger increases in rates of population decline and population growth rate variability when compared to larger‐bodied species.
5. Our results reaffirm and extend our understanding of extinction dynamics in real‐life extirpated populations. In particular, we suggest that smaller‐bodied species may be at greater risk of rapid collapse to extinction than larger‐bodied species, and thus, management of smaller‐bodied species should focus on maintaining higher population abundances as a priority.

## Graphical abstract

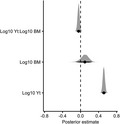

## 1 INTRODUCTION

The Anthropocene is characterized by an unprecedented rate of biodiversity loss driven by a number of anthropogenic stressors including climate change, pollution, habitat loss, overexploitation, and the spread of invasive species (Maxwell et al., 2016; Young et al., 2016). Together, these stressors are reported to have resulted in a 68% decline in vertebrate populations worldwide (WWF, 2020) and a 100‐ to 1,000‐fold increase in the rate of extinction (Barnosky et al., 2011; Ceballos et al., 2015). As populations decline in the face of these stressors, the need for conservation intervention becomes increasingly important to prevent local extinction.

Unabated population decline will eventually result in critically small population sizes, increasing the risk of extirpation through a combination of detrimental genetic, demographic, and environmental processes. The interaction between all these processes may lead to self‐reinforcing, catastrophic downward spirals toward extirpation known as “extinction vortices” (Gilpin & Soulé, 1986), during which there may be little prospect of the population recovering even with intense conservation effort (Palomares et al., 2012). Specifically, per capita growth rates of a population are expected to decline at smaller population sizes, due to a reduction in the fitness of individuals in the population (i.e., the total number of offspring produced by an individual in their lifetime)—a phenomenon known as the “Allee effect” (Berec et al., 2007; Stephens et al., 1999). One mechanism that results in an Allee effect is the loss of genetic diversity due to inbreeding depression (Blomqvist et al., 2010), reducing population growth rates (Bozzuto et al., 2019), and ultimately increasing the risk of extinction (Saccheri et al., 1998). Different forms of stochasticity, causing erratic swings in population growth and decline, also become more important at small population sizes where declines in population growth rates could lead to extinction (Lande, 1993). Demographic stochasticity refers to random fluctuations in population size caused by individual deviations from expected per capita population growth rate and is expected to increase in inverse proportion to population size (Caughley, 1994; Lande, 1993). Moreover, natural fluctuations in external conditions, termed environmental stochasticity, can negatively affect population dynamics by reducing the availability of resources. Indeed, the direct eradication of all extant individuals in a population can be brought about by major environmental disturbances (i.e., catastrophes) such as avalanches, floods, and forest fires (Caughley, 1994).

Several preexisting hypotheses of extinction vortex theory were empirically corroborated by Fagan and Holmes (2006) with a small number of population extirpations, specifically that (a) time to extinction scales with population size on the log scale, indicating that as a population declines the manifold stressors of the extinction vortex are exacerbated and the extinction proneness of the population is elevated, (b) a deterioration in population dynamics occurs (i.e., population growth rate becomes increasingly negative), attributable to declining individual fitness, and (c) variability in population growth rate increases closer to extinction, attributable to an increasing prevalence of stochasticity. However, despite the compelling evidence of extinction vortex dynamics found in real‐life populations (Fagan & Holmes, 2006), we know little about how these extinction dynamics vary according to species' biological traits.

Intrinsic traits may serve as useful predictors of extinction dynamics (Clements et al., 2017; Clements & Ozgul, 2016), with previous work demonstrating that the different strategies employed by taxa to achieve demographic resilience—the ability of a population to withstand and respond to demographic perturbations (Capdevila, Stott, et al., 2020)—are related to components of their life history. For instance, longer lived species seem to be less susceptible to fluctuations in population size from demographic (Sæther et al., 2004, 2013) and environmental (McDonald et al., 2017; Morris et al., 2008; Paniw et al., 2018; Sæther et al., 2013) stochasticity. The presence of persistent adult stages allows long‐lived species to buffer year‐to‐year variability in their vital rates (i.e., fecundity and survival) (Forcada et al., 2008; Morris et al., 2008). Greater susceptibility to stochasticity implies that populations of faster‐living species can be abruptly reduced to a point where the risk of extinction is acutely high. Indeed, short‐lived species have been suggested to be more vulnerable to extinction than long‐lived ones (Jeppsson & Forslund, 2012; Sæther et al., 2005).

The pertinence of understanding how extinction dynamics may vary according to life‐history traits for the management of threatened populations is illustrated by comparing two threatened species on the IUCN Red List: the Javan rhino (*Rhinoceros sondaicus*) and Santa Catarina's guinea pig (*Cavia intermedia*). Both species are listed as critically endangered due to their vanishingly small population sizes (fewer than 50 mature individuals) (Ellis & Talukdar, 2020; Roach, 2016) and are therefore vulnerable to the extinction vortex. However, given the significant differences in their intrinsic traits, for example, a difference in body size spanning around 4 orders of magnitude, if these attributes predict the speed and severity of extinction vortices, it could serve as a useful tool for prioritizing which species to divert conservation effort toward. Whether variation in a specific trait can serve as a useful predictor for how a population responds to the extinction vortex has recently been investigated by Godwin et al. (2020) in a microcosm experiment, finding that populations with stronger sexual selection are more robust to the manifold stressors of the extinction vortex. However, similar studies have not been carried out on natural population data, or with traits which are readily compared across different species.

One trait known to be a good predictor of species' extinction risk is adult body size (henceforth body size) (Ripple et al., 2017). Body size is arguably one of the most important biological traits, as it is associated with a myriad of other intrinsic life‐history attributes, including longevity and rates of reproduction (Brown et al., 2004; Gaillard et al., 1989), which are directly relevant to how a populations' dynamics might change while in the extinction vortex. Furthermore, the relative ease in obtaining body size data for a large number of taxa (Etard et al., 2020) means that comparative studies across species are highly feasible (Capdevila, Beger, et al., 2020; Healy et al., 2019). Indeed, this has elicited an interest in identifying robust and generalizable “rules‐of‐thumb” in conservation based on body size, for example, with respect to setting equitable population targets across species (Hilbers, Santini, et al., 2016). If body size were to act as a predictor of how a population would respond when in the extinction vortex, it could provide a useful tool for making rapid, informed decisions (Clements et al., 2011).

Here, we assess whether body size interacts with underlying demographic processes to influence the dynamics of a population declining toward extinction. We build upon the analyses of Fagan and Holmes (2006), by analyzing data from populations that had been monitored to extinction, as for these populations there is no need to designate quasi‐extinction thresholds, which can result in erroneous interpretation of extinction dynamics. We identified vertebrate populations in which extinction had been observed and combined the resulting time‐series data with information on species‐specific mean body size. We performed three analyses using Bayesian hierarchical models to predict the years to extinction, population growth rates, and population variability, with a particular focus on the effect of body size. We find support for the three aforementioned predictions of the extinction vortex (Fagan & Holmes, 2006). We also find that body size could be an important predictor of the dynamics of populations declining toward extinction, among an ecologically diverse range of species.

## 2 MATERIALS AND METHODS

### 2.1 Population time‐series data

We obtained time‐series data of populations monitored to extirpation from two sources: (a) the Living Planet Database (LPD) (www.livingplanetindex.org/data_portal), a global database containing annual population abundance data for over 25,000 vertebrate populations between 1950 and 2019, and (b) previously published work on the extinction vortex (Fagan & Holmes, 2006). A diverse range of methods to monitor population abundance is included in the LPD. In some cases, complete censuses of the population were carried out, whereas in others population abundance was monitored indirectly. However, the caveat for inclusion of time series in the LPD is that monitoring should be reputable, appropriate for the species, and consistent through time. A more detailed outline of inclusion criteria for the populations in the LPD is provided by Loh et al. (2005).

For inclusion in our analyses, we selected time series that declined to extirpation, which we defined as a population declining to a zero‐abundance observation at the end of the time series. To ensure the ecological relevance of the populations that we included in the study, we excluded time series consisting of aggregated country‐wide abundance data. Zero‐abundance observations occurring before the end of the time series might indicate a relatively low species detectability and, correspondingly, a high rate of observation error (Brook et al., 2006). Therefore, to avoid inflating annual variation in population abundance, we omitted time series where zero observations occurred and were followed by subsequent nonzero observations. Also, to maximize the robustness of our dataset, we only included time series that satisfied all of the following criteria: (a) populations with at least five observations of population abundance prior to extirpation; (b) populations with more than one terminal zero observation; (c) populations where the time between the final nonzero‐abundance observation and the first zero‐abundance observation was no more than one year, so that we could ascertain the exact year in which the population went extinct; and (d) time series covering at least 10 years from the first observation to extirpation, to avoid introducing bias from excessively short time series.

Based on these filtering criteria, we obtained a dataset of 35 populations of 32 different species, including two reptiles, five mammals, and 25 birds (Table S1). The individual time series in the dataset had a minimum and maximum length of 10 and 48 years from the start of the time series to the first zero observation, respectively (mean = 17.40 ± 7.57). Ten of the time series were missing data (i.e., population abundance data were missing from 7.14% to 81.63% of years in the length of the time series).

### 2.2 Body size data

We extracted data on species‐specific mean adult body mass in kg for all species in the above‐detailed dataset from the amniote life‐history database (Myhrvold et al., 2015). In line with other work (Green et al., 2020), we log‐transformed (base 10) the body mass data to improve model fit.

### 2.3 Phylogenetic data

To account for the relatedness of the species included in our analyses, we constructed a species‐level phylogenetic tree with data from the Open Tree of Life (OTL, https://tree.opentreeoflife.org). The OTL combines publicly available taxonomic and phylogenetic information across the tree of life (Hinchliff et al., 2015). Briefly, we built the taxonomic tree using the “rotl” R package (Michonneau et al., 2016). To account for the phylogenetic distance of the species included, we computed the branch length of the resulting tree using the “compute.brlen” function from the R package “ape” (Paradis et al., 2004), with Grafen's arbitrary branch lengths (Grafen, 1989). Polytomies (i.e., a node in the tree with >2 species with a common immediate ancestor) were resolved using the “multi2di” function from the “ape” package (Paradis et al., 2004).

### 2.4 State‐space models

A diverse range of survey methodologies was utilized to monitor the populations included in this study (Table S1). Therefore, to standardize the population trends, we used state‐space models to model abundance through time. State‐space models correct for process noise (*σ* ^2^) and observation/measurement error (*τ* ^2^), both of which are inherent to population time‐series data (Ahrestani et al., 2013). To permit their use in the same analyses, before applying the state‐space model, we scaled population abundance for each year within each time series between 0 and 1. We used the state‐spaced model from Humbert et al. (2009), which takes the form:

(1) $$X_{t}=X_{t‐1}+\mu +\varepsilon_{t}$$

where *X_t_* and *X_t_*_‐1_ are the observed (scaled) abundance estimates in the present (*t*) and past (*t*−1) year, *μ* is the population trend whereby a value of zero represents no change in population abundance and *ε_t_* is the process noise, where

(2) $$\varepsilon_{t}∼gaussian\left(0,\sigma^{2}\right)$$

The errors in observation were added to each *X_t_*:

(3) $$Y_{t}=X_{t}+F_{t}$$

where *Y_t_* is the estimate of the true population abundance and the observation error was:

(4) $$F_{t}∼\text{gaussian}\left(0,\tau^{2}\right)$$

We substituted our estimate of *Y_t_* into [1]:

(5) $$Y_{t}=X_{t‐1}+\mu +\varepsilon_{t}+F_{t}$$

Given

(6) $$X_{t‐1}=Y_{t‐1}‐F_{t‐1}$$

Then:

(7) $$Y_{t}=Y_{t‐1}+\mu +\varepsilon_{t}+F_{t}‐F_{t‐1}$$

### 2.5 Statistical analysis

To investigate the joint effects of body size and abundance or time to extinction on the dynamics of populations prior to extinction, we used Bayesian hierarchical models. To account for the lack of independence among the species included in our analyses, we used the phylogenetic tree to construct a covariance matrix of the species and included it as a random effect. We also included species as a random effect to account for any specific effect that would be independent of the phylogenetic relationship between species (e.g., environmental/niche effects). Prior to running our models, we standardized the covariates to a mean of zero and a standard deviation of one. The general structure of our models was:

(8) $$\mu_{i,j}=\beta_{0}\text{Phylogeny}+\beta_{0}\text{Species}+\beta_{1}\text{Factor}$$

where *i* is a given time series, *j* is a given species, *β*_0_ represents intercepts, *β*_1_ represents slopes, and Factor represents the different fixed effects that we tested. We used weakly informed priors:

(9) $$\beta_{0}\text{Phylogeny}∼\text{Normal}\left(0,1\right)$$

(10) $$\beta_{0}\text{Species}∼\text{Normal}\left(0,1\right)$$

(11) $$\beta_{1}\text{Factor}∼\text{Normal}\left(0,1\right)$$

(12) σ∼Exponential(1)

We ran each model with four chains and for 10,000 iterations, with a warm‐up of 1,000 iterations. We assessed convergence (a) by visually examining the trace plots to ensure that the chains were “well mixed” and (b) using potential scale reduction factor (Rhat) values (i.e., the ratio of the effective sample size to the overall number of iterations), such that values close to one indicate convergence (Table S2). We assessed the importance of our fixed effects according to the position of the credible interval with respect to zero. Specifically, when the 95%, 90%, or 80% credible interval (CI) was larger/smaller than zero, we interpreted this as strong, moderate, and weak evidence for the observed trend, respectively. We have provided a detailed outline of how the structures of the models fit to the three different response variables in their respective sections below. To measure the influence of the phylogeny on our models (phylogenetic signal), we used the posterior distribution of the species variance–covariance matrix (Hadfield & Nakagawa, 2010). A posterior distribution close to zero would indicate a low phylogenetic signal.

#### 2.5.1 Years to extinction

Firstly, we assessed to what degree a species' population size and body size predicted proximity to extinction. To make each time series compatible in the same analyses, we converted time to count backward from extinction to produce a new variable (“*years to extinction*”) with a consistent meaning across all populations. We fitted models, using years to extinction as the response variable and standardized abundance (i.e., *Y_t_* from Equation 7), logged body mass, and their interaction as fixed effects. According to extinction vortex theory, genetic and demographic factors such as inbreeding and demographic stochasticity are exacerbated by diminishing population size. Therefore, time to extinction is expected to change curvilinearly with population size (Lande, 1993), with the model with logged abundance expected to provide a better fit (Fagan & Holmes, 2006). We fitted two independent models with and without a log_10_ transformation on abundance, with a negative binomial distribution of errors. Following Fagan and Holmes (2006), we also excluded the final year of each population from the analysis. The structures of the models were as follows:

(13) $$\text{years}\;\text{to}\;\text{extinction}\;∼Y_{t}+\log_{10}\left(\text{body}\;\text{mass}\right)+Y_{t}:\log_{10}\left(\text{body}\;\text{mass}\right)+\left(1|\text{Phylogeny}\right)+\left(1|\text{Species}\right)$$

(14) $$\text{years}\;\text{to}\;\text{extinction}∼\log_{10}\left(Y_{t}\right)+\log_{10}\left(\text{body}\;\text{mass}\right)+\log_{10}\left(Y_{t}\right):\log_{10}\left(\text{body}\;\text{mass}\right)+\left(1|\text{Phylogeny}\right)+\left(1|\text{Species}\right)$$

To compare the predictive performance of each model, we used expected log predictive density (ELPD) in the “loo” package (Vehtari et al., 2020). If the difference in ELPD is greater than the standard error of the difference, it can be inferred that the predictive performance of each model is different.

#### 2.5.2 Population growth rate

According to the extinction vortex, as a consequence of declining individual fitness due to genetic deterioration and Allee effects, the annual rate of population change is expected to become increasingly negative as population size diminishes. We derived estimates of annual rate of change (*γ_t_*) for each population across the time period:

(15) $$\ln\left(P_{t}\right)=\ln\left(P_{t‐1}\right)+\mu +\varepsilon_{t}$$

(16) $$\gamma_{t}=\ln\left(P_{t}\right)‐\ln\left(P_{t‐1}\right)=\mu +\varepsilon_{t}$$

where *ε_t_* is the process noise and *P_t_* is the smoothed estimate of population abundance. *P_t_* was calculated using a Kalman filter that uses parameters derived from the state‐space model to correct for both measurement error (*τ* ^2^) and variance (*v* ^2^) (Humbert et al., 2009; Leung et al., 2017). We fitted a model with the structure:

(17) $$\gamma_{t}∼\text{years}\;\text{to}\;\text{extinction}+\log_{10}\left(\text{body}\;\text{mass}\right)+\text{years}\;\text{to}\;\text{extinction}:\log_{10}\left(\text{body}\;\text{mass}\right)+\left(1\text{|Phylogeny}\right)+\left(1\text{|Species}\right)$$

A positive coefficient for years to extinction in this model would support the hypothesis that per capita growth rate decreases closer to extinction.

#### 2.5.3 Residual variability

As populations decline, the influence of stochasticity is expected to increase and contribute to their extinction. This should manifest in greater annual variability in population change at closer proximity to extinction. To avoid removing the underlying pattern of demographic stochasticity, we did not transform the population data using state‐space models for this analysis. Instead, to quantify annual variability in population growth rate, we calculated geometric growth rate (*r*) as:

(18) r=ln(λ)

(19) $$\lambda =N_{t}/N_{t+1}$$

where *N_t_* is the raw population abundance in a given year and *N_t_*_+1_ is the raw population abundance in the previous year. As the logarithm of zero is not resolvable, we could not obtain finite estimates of a populations' final growth rate before extirpation. We subsequently extracted the residuals from a model of the structure:

(20) r∼yearstoextinction+(1|Phylogeny)+(1|Species)

and squared them (residuals^2^). We fitted a model with the structure:

(21) $${\text{residuals}}^{2}∼\;\text{years}\;\text{to}\;\text{extinction}\;+\log_{10}\left(\text{body}\;\text{mass}\right)+\;\text{years}\;\text{to}\;\text{extinction}:\log_{10}\left(\text{body}\;\text{mass}\right)\left(1|\text{Phylogeny}\right)+\left(1|\text{Species}\right)$$

Support for the hypothesis that variability in annual population growth rate increases as extinction draws nearer in time would be found by a negative coefficient for years to extinction in this model.

We carried out all statistical analyses using R v4.0.0 (R Core Team, 2020) and used the “brms” package v2.1.0 (Bürkner, 2018) to fit our models. In the Supporting Information, we have also provided (a) a complete summary of the populations used in this dataset (Table S1), (b) figures faceted by species to show how the models fit to each species (Figures S1–S3), (c) a figure showing the linear relationship between body mass and other quantitative life‐history traits (Figure S4), and (d) a table showing the sample sizes and output summary of the models from each analysis (Table S2).

## 3 RESULTS

### 3.1 Years to extinction

**FIGURE 1:**
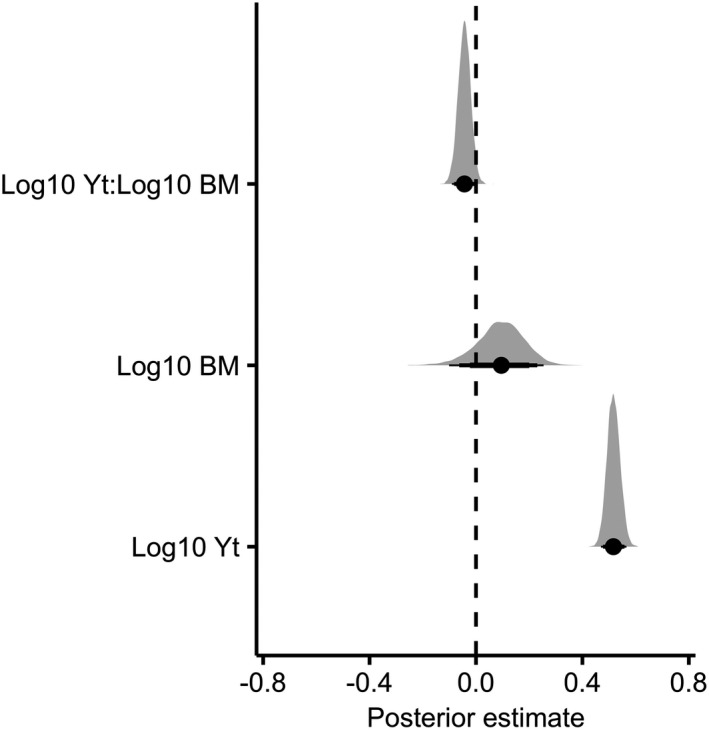
Coefficient plot showing posterior estimates of fixed effects for the first analysis (*years to extinction*). Dashed vertical line shows the zero slope, whereby there is no effect of the fixed effects. Each density plot is based on 1,000 samples from the posterior distribution of the parameter estimates. Reported values are the highest posterior density median values (filled circles), with 80% (thickest bars), 90%, and 95% credible intervals. Yt = population abundance and BM = body mass

In agreement with the hypothesis that populations exhibit a nonlinear decline to extinction, based on ELPD the hierarchical models with logged abundance (ELPD difference = 0.0; *SE* = 0.0) provided a better predictive performance than that with nonlogged abundance (ELPD difference = −28.40; *SE* = 2.90). Intuitively, there was a strong positive relationship between logged population abundance and years to extinction (median = 0.52; 95% CIs [low =0.47; high = 0.57]; Figure 1). There was also moderate evidence for a negative interaction between logged population abundance and logged body size (median = −0.044; 95% CIs [low = −0.087; high = 0.0011]; Figure 1), suggesting that the speed in which populations collapse to extinction has a tendency to be slower for larger‐bodied species. There was a weak phylogenetic signal in these models (mean ± *SE* 0.36 ± 0.24), meaning that evolutionary history could only explain a fraction of the observed variability in the patterns of abundance decline with years to extinction.

### 3.2 Population growth rate

**FIGURE 2:**
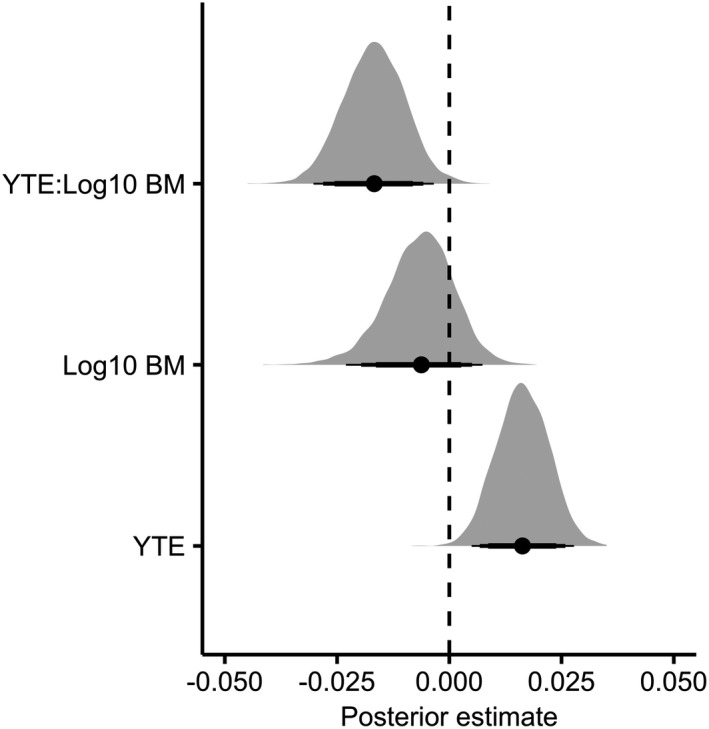
Coefficient plot showing posterior estimates of fixed effects for the second analysis (*population growth rate*). Dashed vertical line shows the zero slope, whereby there is no effect of the fixed effects. Each density plot is based on 1,000 samples from the posterior distribution of the parameter estimates. Reported values are the highest posterior density median values (filled circles), with 80% (thickest bars), 90%, and 95% credible intervals. YTE = Years to extinction and BM = body mass

We found a positive relationship between geometric growth rate and years to extinction in the model (median = 0.016; 95% CIs [low = 0.0050; high = 0.028]; Figure 2), supporting the hypothesis that a deterioration in population dynamics occurs during the decline to extirpation. We found no evidence for an effect of logged body size on population growth rates (median = −0.0062; 95% CIs [low = −0.023; high = 0.0074]; Figure 2); however, there was strong evidence for a negative interaction between years to extinction and logged body size (median = −0.017; 95% CIs [low = −0.030; high = 0.0035]; Figure 2), suggesting that the negative relationship between geometric growth rate and years to extinction is weaker in larger‐bodied species. Finally, there was a moderate phylogenetic signal in this model (0.65 ± 0.34), implying that evolutionary history played a minor role in explaining the differences among the different studied groups.

### 3.3 Residual variability

**FIGURE 3:**
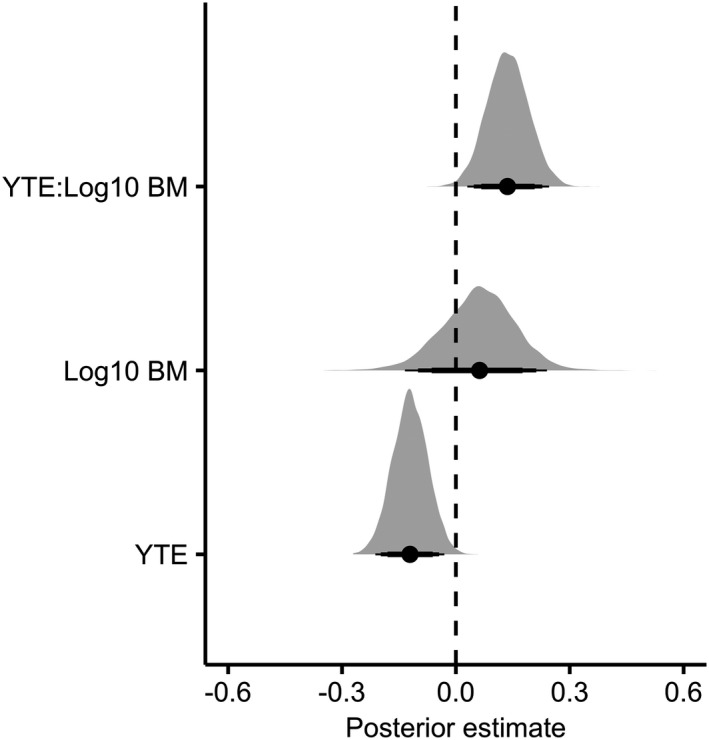
Coefficient plot showing posterior estimates of fixed effects for the third analysis (*residual variability*). Dashed vertical line shows the zero slope, whereby there is no effect of the fixed effects. Each density plot is based on 1,000 samples from the posterior distribution of the parameter estimates. Reported values are the highest posterior density median values (filled circles), with 80% (thickest bars), 90%, and 95% credible intervals. YTE = Years to extinction and BM = body mass

We found strong evidence for a negative relationship between residual variability and years to extinction in the model (median = 0.45; 95% CIs [low = 0.24; high = 0.66]; Figure 3), supporting the hypothesis that there is an increase in the prevalence of stochasticity influencing populations closer to extinction. There was no evidence for an effect of logged body size on the magnitude of residual variability (median = 0.060; 95% CIs [low = −0.087; high = 0.22]; Figure 3). However, there was strong evidence for a positive interaction between years to extinction and logged body size (median = 0.14; 95% CIs [low = 0.050; high = 0.23]; Figure 3), suggesting that the negative relationship between residual variability and years to extinction is weaker in larger‐bodied species. Besides, there was a weak phylogenetic signal in this model (0.25 ± 0.24), meaning that evolutionary history could only explain a small fraction of the variability in the model.

## 4 DISCUSSION

Understanding the dynamics of small populations is critical for the effective conservation of at‐risk species. Previous work has demonstrated that the dynamics of natural populations declining toward extinction conform to predictions derived from extinction vortex theory, such that (a) time to extinction scales logarithmically with population size, (b) the rate of population decline increases closer to extinction, and (c) residual variability increases closer to extinction (Fagan & Holmes, 2006). However, empirical studies investigating how intrinsic traits influence extinction dynamics have been lacking. We assembled a dataset of time series representing populations monitored to extinction and performed three analyses to test whether a key ecological trait—body size—is an important predictor of the dynamics of populations declining toward extinction. Our results show that body size is an important predictor of extinction dynamics and also reaffirm previous empirical findings supporting the existence of extinction vortex dynamics in real extirpated populations.

Given that the mutually reinforcing negative effects of the extinction vortex are exacerbated by diminishing population size, the extinction proneness of a population is expected to increase as its size diminishes (Fagan & Holmes, 2006). Therefore, years to extinction is expected to scale better to the logarithm of population size (Lande, 1993). Our results support the hypothesis that the proximity of a population to extinction is dependent on the logarithm of population size, indicative of an extinction vortex. Accordingly, care should be taken to maintain large population sizes to avoid self‐reinforcing spirals to extinction and to maximize the probability of long‐term persistence.

The result of our first analysis, showing moderate evidence for a negative interaction between population abundance and body size in determining time to extinction (Figure 1), indicates that larger‐bodied organisms have a tendency to collapse to extinction more slowly. Although it is increasingly acknowledged that the level of extinction risk (e.g., IUCN Red List category) is an emergent property of the interaction between biological traits and the type of threatening process (Davidson et al., 2009; Isaac & Cowlishaw, 2004; Owens & Bennett, 2000; Price & Gittleman, 2007; Ripple et al., 2017), this result may seem at odds with the frequently reported positive association between body size and extinction risk (Dirzo et al., 2014; Gaston & Blackburn, 1995; Hilbers, Schipper, et al., 2016). However, we argue that this can be explained by allometric‐related differences in life history and resilience across species. Body size scales with pace of life such that larger‐bodied species have greater longevity and older ages at maturity and produce fewer offspring per year (Figure S4). The susceptibility of growth rates of slower‐living organisms to fluctuating environmental conditions is generally smaller (McDonald et al., 2017; Morris et al., 2008; Paniw et al., 2018; Sæther et al., 2013) due to the prioritization in survival over reproduction (Morris et al., 2008). The influence of demographic stochasticity on population dynamics has also been shown to be smaller in species with slower life histories (Sæther et al., 2004, 2013). As such, species with these traits are buffered against abrupt, drastic reductions in population size, drawing out extinction over a longer period of time (Jeppsson & Forslund, 2012; Sæther et al., 2005). Indeed, while the extinction risk of highly fecund species is tempered by naturally larger population growth rates (Brook & Bowman, 2005; Hilbers, Schipper, et al., 2016); smaller‐bodied and faster‐living species appear to be more threatened with extinction after correcting for the confounding effect of population size (Hilbers, Santini, et al., 2016; Jeppsson & Forslund, 2012; Johst & Brandl, 1997; Newmark, 1995; Sæther et al., 2005).

According to extinction vortex theory, genetic deterioration and Allee effects are expected to result in proportionally larger declines as population size diminishes. Indeed, the result of our second analysis suggests that the key question of when a species is at risk of rapidly collapsing to extinction is a function of population size, with an increase in the year‐to‐year rate of decline at closer proximity to extinction (Figure 2). The implication of this is that even with conservation intervention, species that fall into the extinction vortex may struggle to be saved and require a nonlinear increase in the magnitude of the intervention required to save a population as it moves toward extinction. Well‐studied populations on the verge of extirpation support this. For example, the decline of the Florida panther population (*Puma concolor coryi*) was only reversed after the introduction of several individuals translocated from healthy populations leading to the restoration of genetic diversity (Johnson et al., 2010). In practical terms, this emphasizes the need for early conservation intervention, with a strong focus on ensuring populations do not fall into the extinction vortex.

We found evidence that the deterioration in population dynamics observed in populations declining toward extinction was more pronounced in species with smaller body size (Figure 2). We believe this can also be accounted for by disparate life‐history strategies among species with different body sizes. Overall, by spreading their reproductive effort over many years, long‐lived species reduce the impact of reproductive failure over a given unit of time (Morris et al., 2008). As such, slower paces of life, as observed in larger‐bodied species likely provides a means of delaying the impact of deleterious processes such as Allee effects and demographic stochasticity, on their demographic rates.

Demographic stochasticity increases in inverse proportion to population size (Caughley, 1994) and is expected to contribute to the extinction of dwindling populations (Brook et al., 2008). The results of our third analysis, demonstrating an increase in annual residual variability of population growth rate at closer proximity to extinction (Figure 3), support the hypothesis that stochastic processes contributed to the extirpation of these populations. Importantly, demographic stochasticity is purported to be a major factor inhibiting the recovery of well‐studied threatened populations, despite substantial conservation effort (Palomares et al., 2012).

We also found evidence that the rate in which fluctuations in population dynamics are magnified closer to extirpation is greater among smaller‐bodied species (Figure 3); in other words, stochasticity seems to assume greater importance as a contributing factor to the extinction of populations for smaller‐bodied species. The influence of stochastic elements on demographic variance is expected to be greater in faster‐living species (Sæther et al., 2013). Accordingly, the risk of extinction induced by stochasticity is generally higher for faster‐living species (Jeppsson & Forslund, 2012; Sæther et al., 2005).

The cumulative evidence herein indicates that body size may, in fact, serve as a good predictor of population dynamics during the final decline to extirpation. Specifically, by displaying a steeper decline to extinction, greater deterioration in population dynamics and a greater increase in residual variability closer to extirpation, we find that life histories associated with small body size might have an exacerbating effect on extinction vortex. Accordingly, both the intensity and urgency of conservation intervention required might be greater for smaller‐bodied species, upon reaching small population sizes and entering the extinction vortex. Nevertheless, given the relatively short timescales covered by these time series and that the ultimate fate of all populations in this study was extinction, our study does not provide a complete picture of how body size influences the extinction risk of species, per se. For example, we are unable to quantify the population size threshold below which extirpation is inevitable and whether this differs between species according to body size (Hilbers, Santini, et al., 2016). Moreover, we could not assess whether conservation activities are effective in saving populations that have fallen into an extinction vortex. In fact, it is possible that efforts to conserve a population in the extinction vortex merely serves to temporarily postpone extirpation (Palomares et al., 2012), suggesting that it might be more prudent to divert resources toward less threatened populations with better survival prospects.

The relatively weak phylogenetic signal in our models suggests that the relationships we observed between the dynamics in populations declining toward extinction and our fixed effects were not primarily determined by the evolutionary history of the species. However, there was a major phylogenetic bias in our dataset with Aves, overwhelmingly, being the most well‐represented class (i.e., 71.43% of populations were avian). This may have important implications for the interpretation of our results. In general, the extinction risk imposed by demographic stochasticity is expected to decline at lower fecundities, with the exception of very short‐lived species where the opposite is expected (Jeppsson & Forslund, 2012). Indeed, birds are relatively long‐lived compared to other taxa (Healy et al., 2014), which might explain why slow life‐history traits have been associated with greater persistence among invading populations of birds (Sol et al., 2012), but not other taxa (Allen et al., 2017; Capellini et al., 2015). Therefore, the preponderance of avian species in our dataset may have biased our results. Furthermore, our dataset consisted almost entirely of terrestrial species (Table S1), preventing us analyzing how habitat type might modulate the severity of extinction vortices. Differences in temporal environmental autocorrelation between terrestrial and aquatic systems (Dawson & Hamner, 2008) mean that the selection pressures in the two systems are not the same (Steele et al., 2019), as reflected by the divergent life‐history strategies between terrestrial and aquatic species (Capdevila, Beger, et al., 2020). Although regrettable from a conservation standpoint, with the potential of revealing important differences between taxonomic classes and environmental systems, it would be interesting to perform similar analyses when a larger dataset of population extirpations becomes available.

To conclude, we investigated demographic response to the extinction vortex in relation to an important intrinsic trait, in real‐life extirpated populations. We demonstrate the existence of extinction vortex dynamics using a larger dataset of population extirpations than previous work (Fagan & Holmes, 2006). We also find that body size might be an important predictor of population dynamics prior to extirpation. However, given the nature of our relatively small dataset, with sparse representation across time, space, and phylogeny, our results should be viewed as a preliminary insight into how extinction dynamics vary according to intrinsic traits. At present, we emphasize that preserving sufficiently high population sizes should be by far the most important consideration in order to safeguard threatened taxa from extinction.

## CONFLICTS OF INTEREST

The authors declare no conflict of interest.

## AUTHORS' CONTRIBUTIONS

C.C.: Conceptualization of the idea with inputs from all authors. N.W.: Data assemblage. All authors designed the methodology. N.W. and P.C.: Analyses. N.W.: Writing of the manuscript. All authors contributed critically to the drafts and gave final approval for publication.

## Supporting information

Supplementary Material

## Data Availability

The Living Planet Database is available at: www.livingplanetindex.org/data_portal. The data and code supporting the results have been deposited in the Dryad Digital Repository https://doi.org/10.5061/dryad.jwstqjq8c.
