# Supplementary material for: Scaling the extinction vortex: Body size as a predictor of population dynamics close to extinction events
Source: Ecol Evol. 2021 May 2;11(11):7069–79. doi: 10.1002/ece3.7555 (PMC8207159; doi:10.1002/ece3.7555)
Supplement: Supplementary file 1 — Supplementary Material [file ECE3-11-7069-s001.docx]

**Electronic supplementary material**

# Scaling the extinction vortex: Body size as a predictor of population dynamics close to extinction events

Nathan F. Williams^1^*, Louise McRae^2^, Robin Freeman^2^, Pol Capdevila^1^, Christopher F. Clements^1^

**Affiliations:**

^1^School of Biological Sciences, University of Bristol, Bristol, BS8 1TQ, UK

^2^Institute of Zoology, Zoological Society of London, London, NW1 4RY, UK

**Author email addresses:** [nathan.williams.mail@gmail.com](mailto:nathan.williams.mail@gmail.com); [louise.mcrae@ioz.ac.uk](mailto:louise.mcrae@ioz.ac.uk); [robin.freeman@ioz.ac.uk](mailto:robin.freeman@ioz.ac.uk); [pcapdevila.pc@gmail.com](mailto:pcapdevila.pc@gmail.com); [c.clements@bristol.ac.uk](mailto:c.clements@bristol.ac.uk)

***Correspondence to:** Email: [nathan.williams.mail@gmail.com](mailto:nathan.williams.mail@gmail.com)

| **Table S1.** A summary of the populations and species in the dataset. ‘Data type’ refers to the nature of the study and ‘units’ refers to the units of population abundance being measured. | | | | | | |
| --- | --- | --- | --- | --- | --- | --- |
| Population | Species binomial | Common name | Class | Data type | Units | Time series length (years) |
| 1 | *Acanthiza reguloides* | Buff-rumped thornbill | Aves | Sample | Individuals / survey | 10 |
| 2 | *Alces alces* | Elk / Moose | Mammalia | Estimate | Individuals | 17 |
| 3 | *Alces alces* | Elk / Moose | Mammalia | Estimate | Individuals | 12 |
| 4 | *Corvus hawaiiensis* | Hawaiian crow | Aves | Full population count | Individuals | 12 |
| 5 | *Corvus kubyari* | Mariana crow | Aves | Density | Individuals / 100km | 12 |
| 6 | *Dendrocopos medius* | Middle-spotted woodpecker | Aves | Full population count | Individuals | 23 |
| 7 | *Gallicolumba xanthonura* | White-throated ground dove | Aves | Density | Individuals / 100km | 16 |
| 8 | *Glyptemys insculpta* | North American wood turtle | Reptilia | Full population count | Individuals | 19 |
| 9 | *Glyptemys insculpta* | North American wood turtle | Reptilia | Full population count | Individuals | 19 |
| 10 | *Grus americana* | Whooping crane | Aves | Full population count | Individuals | 18 |
| 11 | *Gyps bengalensis* | White-rumped vulture | Aves | Proxy | Active nests | 15 |
| 12 | *Gyps fulvus* | Griffon vulture | Aves | Full population count | Breeding pairs | 14 |
| 13 | *Lanius minor* | Lesser-grey shrike | Aves |  | Breeding pairs | 14 |
| 14 | *Lycaon pictus* | African wild dog | Mammalia | Full population count | Individuals | 23 |
| 15 | *Marmota vancouverensis* | Vancouver Island marmot | Mammalia | Full population count | Individuals | 20 |
| 16 | *Myzomela rubratra* | Micronesian myzomela | Aves | Density | Individuals / 100km | 10 |
| 17 | *Passer domesticus* | House sparrow | Aves | Density | Individuals / km^2^ | 13 |
| 18 | *Pezoporus wallicus* | Eastern ground parrot | Aves | Proxy | Individuals / site | 13 |
| 19 | *Phalacrocorax carbo* | Great cormorant | Aves | Proxy | Breeding pairs | 21 |
| 20 | *Picoides borealis* | Red-cockaded woodpecker | Aves | Full population count | Individuals | 14 |
| 21 | *Pluvialis apricaria* | European golden plover | Aves | Full population count | Individuals | 18 |
| 22 | *Pluvialis fulva* | Pacific golden plover | Aves | Full population count | Individuals | 28 |
| 23 | *Pseudechis australis* | King brown / Mulga snake | Reptilia | Measure per unit effort | Encounter rate | 10 |
| 24 | *Ptilinopus roseicapilla* | Mariana fruit dove | Aves | Density | Individuals / 100km | 11 |
| 25 | *Pygoscelis adeliae* | Adelié penguin | Aves | Estimate | Breeding pairs | 29 |
| 26 | *Pygoscelis antarcticus* | Chinstrap penguin | Aves | Proxy | Breeding pairs | 49 |
| 27 | *Rangifer tarandus* | Caribou / Reindeer | Mammalia | Full population count | Individuals | 21 |
| 28 | *Rangifer tarandus* | Caribou / Reindeer | Mammalia | Full population count | Individuals | 23 |
| 29 | *Rhipidura rufifrons* | Rufous fantail | Aves | Density | Individuals / 100km | 10 |
| 30 | *Sterna dougallii* | Roseate tern | Aves | Proxy | Breeding pairs | 22 |
| 31 | *Struthio camelus* | Common ostrich | Aves | Estimate | Individuals | 13 |
| 32 | *Sturnus vulgaris* | Eurasian starling | Aves | Proxy | Nests | 14 |
| 33 | *Todiramphus cinnamominus* | Guam kingfisher | Aves | Density | Individuals / 100km | 22 |
| 34 | *Urocitellus parryi* | Arctic ground squirrel | Mammalia | Density | Density / ha | 14 |
| 35 | *Vanellus vanellus* | Lapwing | Aves | Sample | Breeding pairs | 10 |


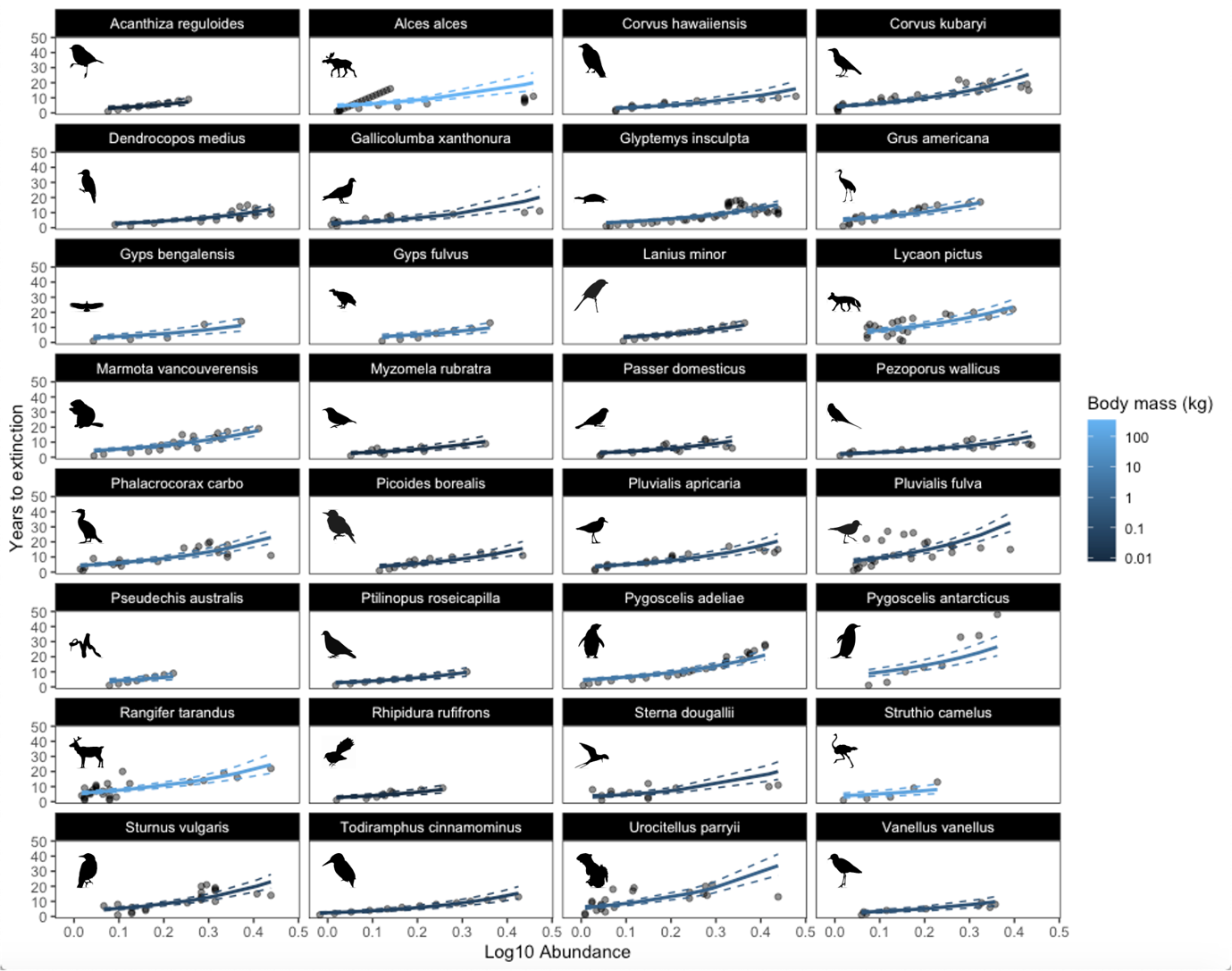


**Figure S1.** Years to extinction against population abundance, faceted by individual population and arranged alphabetically by species name. Within each facet, circles show actual data and lines show estimates from the hierarchical model, coloured according to body size. Dashed lines show upper and lower 95% CIs of the slope. Animal silhouettes were taken from PhyloPic (PhyloPic – Free Silhouette Images of Life Forms*).

***Credit for the animal silhouettes is given to the following:** T. Michael Keesey (*Corvus hawaiiensis, Pezoporus wallicus*); Luc Viatour & Andreas Plank (*Gallicolumba xanthonura*); Scott Hartman (*Glyptemys insculpta*); Shyamal (*Gyps bengalensis*); Gabriela Palomo-Munoz (*Lycaon pictus*); Andre Butko (*Passer domesticus*); Emily Willoughby (*Pluvialis apricaria*); Chris Hay (*Pseudechis australis*); Noah Schlottman (*Pygoscelis adeliae*); Maxime Dahirel (*Sturnus vulgaris*); Rebecca Groom (*Vanellus vanellus*).

No changes were made to the silhouettes. Use of these silhouettes is permitted here under the Creative Commons Attribution-Sharelike 3.0 Unported license: <https://creativecommons.org/licenses/by-sa/3.0/>


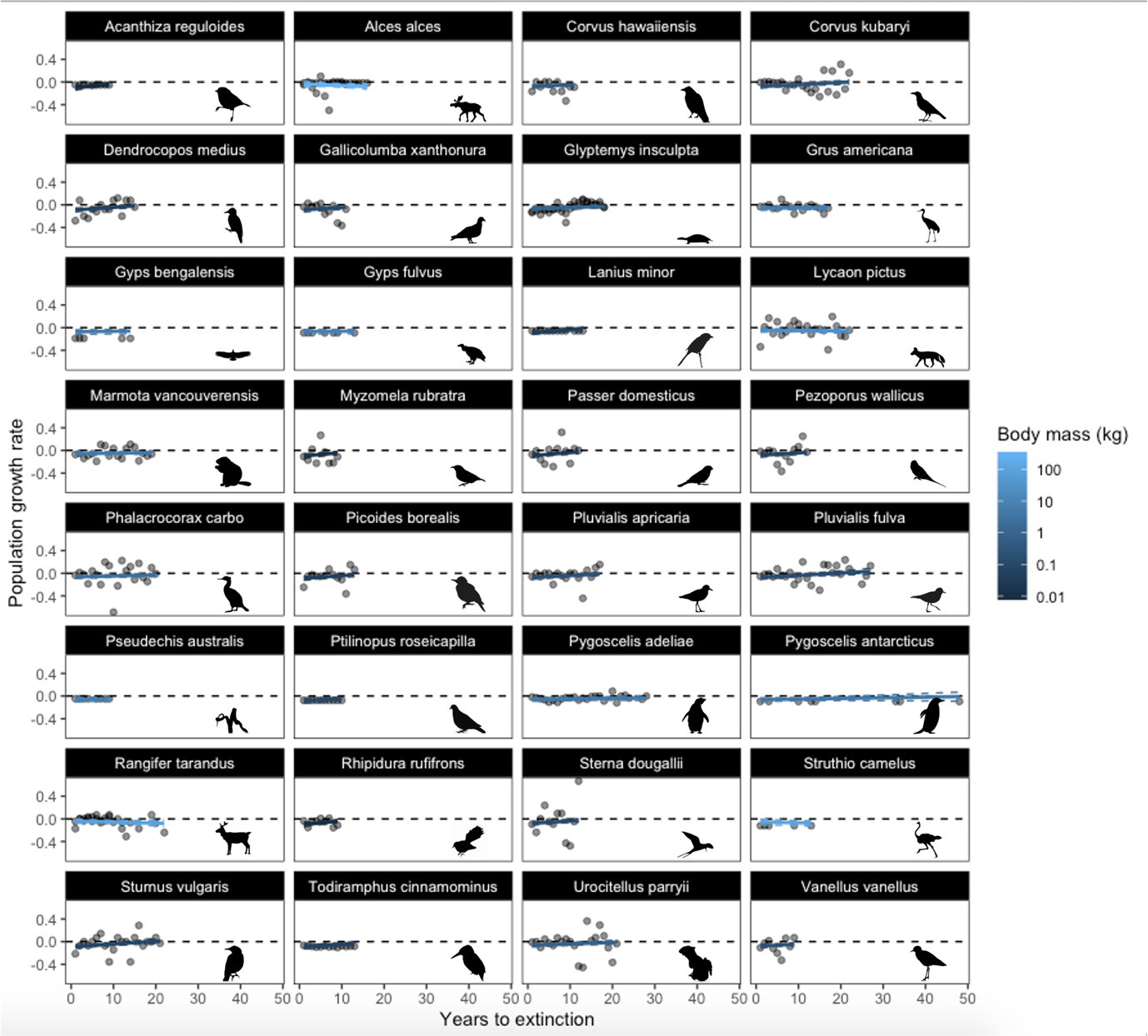


**Figure S2.** Population growth rate against years to extinction, faceted by individual population and arranged alphabetically by species name. Within each facet, circles show actual data and lines show estimates from the hierarchical model, coloured according to body size. Dashed lines show upper and lower 95% CIs of the slope.


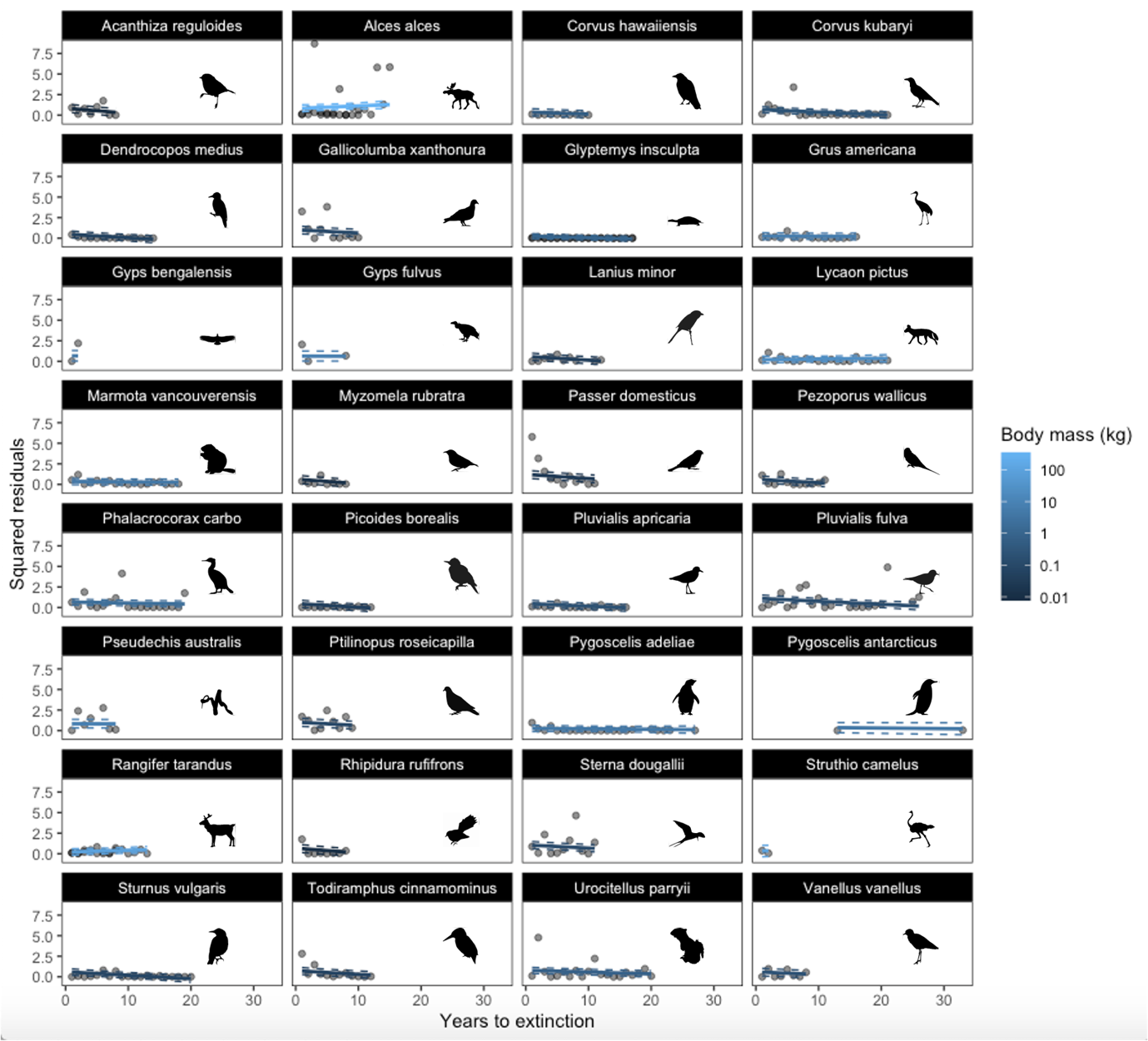


**Figure S3.** Residual variability against population abundance, faceted by individual population and arranged alphabetically by species name. Within each facet, circles show actual data and lines show estimates from the hierarchical model, coloured according to body size. Dashed lines show upper and lower 95% CIs of the slope.

**Figure S4.** Linear relationship between logged adult body mass and three other logged quantitative life history traits, including the regression lines and confidence intervals. Offspring per year was calculated by multiplying average clutch size by number of clutches per year. Data for all traits was extracted from the amniote life history database (Myrhvold et al., 2015). *Note*: data for longevity, age at female maturity and number of offspring per year was not available for nine, eleven and nine species, respectively. This highlights why we focused on body size in the manuscript (i.e., given the broader taxonomic coverage of available data for this trait).

| **Table S2.** A summary of Bayesian hierarchical models from each analysis. Medians and 95% CIs are in bold if the 95% CIs do not overlap with zero. | | | | | |  |
| --- | --- | --- | --- | --- | --- | --- |
| Analysis | Model structure | Fixed | Median (95% CIs) | Rhat | Pd | Sample size |
| **Years to extinction** | | | | | |  |
| *Log_10_(Abundance) + Log_10_(Body mass) + Log_10_(Abundance):Log_10_(Body mass)* | | | | | | 489 |
|  |  | Log_10_(Abundance) | **0.52 (0.47 - 0.57)** | 1.00 | 1.00 |  |
|  |  | Log_10_(Body mass) | 0.10 (-0.10 – 0.25) | 1.00 | 0.86 |  |
|  |  | Log_10_(Abundance):Log_10_(Body mass) | -0.044 (-0.087 – 0.0011) | 1.00 | 0.97 |  |
| *Abundance + Log_10_(Body mass) + Abundance:Log_10_(Body mass)* | | | | | | 489 |
|  |  | Abundance | **0.48 (0.43 – 0.53)** | 1.00 | 1.00 |  |
|  |  | Log_10_(Body mass) | 0.089 (-0.096 – 0.25) | 1.00 | 0.83 |  |
|  |  | Abundance:Log_10_(Body mass) | -0.041 (-0.089 – 0.0072) | 1.00 | 0.95 |  |
| **Geometric growth rate** | | | | | |  |
| *Years to extinction + Log_10_(Body mass) + Years to extinction:Log_10_(Body mass)* | | | | | | 489 |
|  |  | Years to extinction | **0.016 (0.0050 – 0.028)** | 1.00 | 1.00 |  |
|  |  | Log_10_(Body mass) | -0.0062 (-0.023 – 0.0074) | 1.00 | 0.82 |  |
|  |  | Years to extinction:Log_10_(Body mass) | **-0.017 (-0.030** – **-0.0035)** | 1.00 | 0.99 |  |
| **Residual variability**  *Years to extinction + Log_10_(Body mass) + Years to extinction:Log_10_(Body mass)* | | | | | | 432 |
|  |  | Years to extinction | **0.45 (0.24 – 0.66)** | 1.00 | 1.00 |  |
|  |  | Log_10_(Body mass) | 0.060 (-0.087 – 0.22) | 1.00 | 0.74 |  |
|  |  | Years to extinction:Log_10_(Body mass) | **0.14 (0.05 – 0.23)** | 1.00 | 0.99 |  |
| CI: Credible interval; Pd: Probability of direction | | | | | |  |
